# Proactive Approach for Safe Use of Antimicrobial Coatings in Healthcare Settings: Opinion of the COST Action Network AMiCI

**DOI:** 10.3390/ijerph14040366

**Published:** 2017-03-31

**Authors:** Merja Ahonen, Anne Kahru, Angela Ivask, Kaja Kasemets, Siiri Kõljalg, Paride Mantecca, Ivana Vinković Vrček, Minna M. Keinänen-Toivola, Francy Crijns

**Affiliations:** 1 Faculty of Technology, Satakunta University of Applied Sciences, P.O. Box 211, FI-26101 Rauma, Finland; minna.keinanen-toivola@samk.fi; 2 Laboratory of Environmental Toxicology, National Institute of Chemical Physics and Biophysics, Akadeemia tee 23, Tallinn 12618, Estonia; angela.ivask@kbfi.ee (A.I.); kaja.kasemets@kbfi.ee (K.K.); 3 Estonian Academy of Sciences, Kohtu 6, Tallinn 10130, Estonia; 4 Department of Microbiology, Institute of Biomedicine and Translational Medicine, University of Tartu, Ravila 19, Tartu 50411, Estonia; siiri.koljalg@kliinikum.ee; 5 Department of Earth and Environmental Sciences, Research Centre POLARIS, University of Milano-Bicocca, 1 Piazza della Scienza, 20126 Milan, Italy; paride.mantecca@unimib.it; 6 Institute for Medical Research and Occupational Health, Ksaverska cesta 2, Zagreb 10000, Croatia; ivinkovic@imi.hr; 7 Department Bèta Sciences and Technology, Zuyd University of Applied Sciences, P.O. Box 550, 6400 AN Heerlen, The Netherlands; francy.crijns@zuyd.nl

**Keywords:** nanomaterials, silver, copper, safety, healthcare associated infections, ecotoxicity, antimicrobial resistance, risk-benefit analysis

## Abstract

Infections and infectious diseases are considered a major challenge to human health in healthcare units worldwide. This opinion paper was initiated by EU COST Action network AMiCI (AntiMicrobial Coating Innovations) and focuses on scientific information essential for weighing the risks and benefits of antimicrobial surfaces in healthcare settings. Particular attention is drawn on nanomaterial-based antimicrobial surfaces in frequently-touched areas in healthcare settings and the potential of these nano-enabled coatings to induce (eco)toxicological hazard and antimicrobial resistance. Possibilities to minimize those risks e.g., at the level of safe-by-design are demonstrated.

## 1. Introduction

Infectious diseases are an increasing global concern to human health. European Centre for Disease prevention and Control [1] has estimated that over 4 million people are acquiring a HealthCare Associated Infection (HCAI) annually leading to 37,000 deaths as a direct consequence of these infections. HCAIs are considered a major health challenge in healthcare units worldwide, whereas conditions derived from HCAIs are considered the sixth leading cause of death in western countries and even higher in developing countries [2].

The corner stones of maintaining healthy environment in hospitals include appropriate use of disinfectants (cleaning, disinfecting, hand hygiene) and antibiotics. Despite the importance of cleaning and disinfection of frequently-touched surfaces and monitoring the hygiene of these surfaces [3], it has been shown that less than half of the near patient surfaces are regularly cleaned [4,5,6], thus paving the way to HCAIs [7]. Therefore, new approaches are required to reduce microbial activity, associated infections and the development of antimicrobial resistance (AMR).

A potential and promising weapon against bacterial growth in healthcare sector has been found in AntiMicrobial Coatings (AMCs) [8]. Many different chemical strategies and technologies for AMCs have been described: (i) AMCs may contain active eluting agents (e.g., ions or nanoparticles of silver, copper, zinc, or antibiotics, chloride, iodine); (ii) immobilized molecules that become active upon contact (e.g., quaternary ammonium polymers or peptides, chitosan); or (iii) light-activated molecules (e.g., TiO_2_ or photosensitizers) [9,10,11]. Particularly, nanoparticles (NPs), e.g., silver, copper, and zinc NPs, are being more and more often introduced to AMCs due to the growing recognition of their superior biocidal efficacy [12]. As an example, silver [13] and CuO [14,15] have been used as additives in hospital fabrics (face masks, privacy curtains, bedsheets, and other healthcare textiles). AMCs, mostly nanosilver is widely used as an antimicrobial additive in bandages, wound dressing and in urinary and intravenous catheters [16,17] but this usage area of AMCs is out of the scope of the current paper that is focused on frequently-touched surfaces.

**Figure 1:**
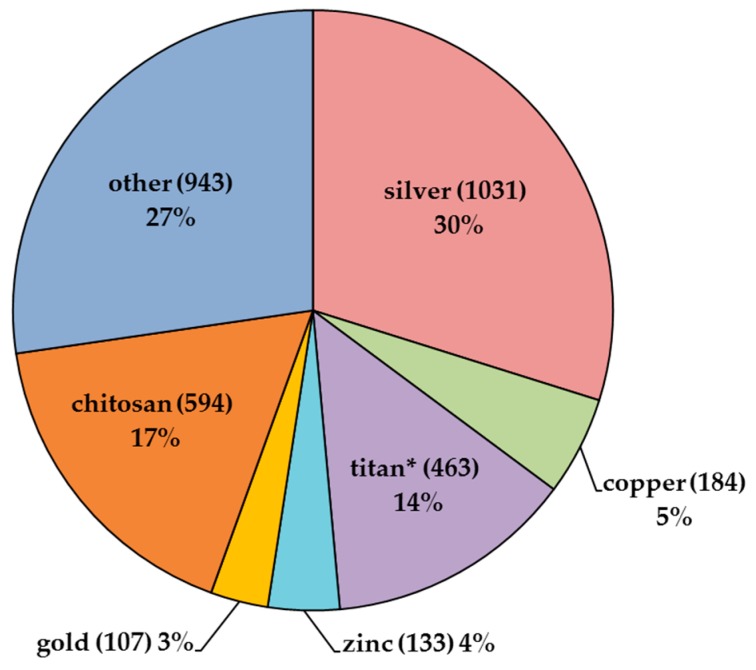
The share of papers in ISI Web of Science (number and %) for different materials with antimicrobial properties within the pool of papers (3455 in sum) described by truncated search term ’antimicrobial coating’. The materials are presented as data labels. The number of papers in the category ‘other’ was calculated by subtracting papers for silver, copper, titanium, zinc, gold and chitosan from the total nr of papers for antimicrobial coatings (3455). The search was performed on 14 March 2017.

The search made in ISI Web of Science using the term ’antimicrobial coating*’ yielded 3455 publications (Figure 1). Within this pool of papers, 1031 hits were obtained for ’silver’, 463 for ’titan*’, 184 for ’copper’, 133 for ’zinc’ and 107 for ’gold’. In a comparative performance assessment of commercially available AMCs, it has been demonstrated that silver NPs are currently the most applied and efficient nanomaterial (NM) in AMCs [18]. It is important to note that not only metallic, but also other types of NPs (e.g., chitosan) can be incorporated to AMCs (594 papers; Figure 1). As the application of those is mostly concerning food industry, these NPs are beyond the scope of the current review that is focused mainly on metal-based antimicrobial compounds frequently applied in healthcare settings.

The data on the efficiency of AMCs application in healthcare settings are relatively scarce. A recent review paper published by Müller et al. [19] showed that there was a limited number of high-quality studies on this issue and the data that passed the evaluation criteria (altogether 11 cases) concerned mostly copper (*n* = 7), silver (*n* = 1), metal-alloy (*n* = 1) and organo-silane (*n* = 1). Other researchers, showed that copper touch surfaces tested in hospital patient room and kindergarten had lower total bacteria and *Staphylococcus aureus* counts compared to non-copper touch surfaces [20]. The application of copper (alloys) also reduced bacterial numbers on frequently-touched surfaces in the intensive care units [21,22] and in hospital patients rooms [19]. Some studies also suggest that the application of copper in patient rooms’ solid touch surfaces [23,24] or copper containing linens [25] may reduce the HCAI compared with non-copper reference surfaces. However, as Boyce [26] has concluded, further studies of the long-term antimicrobial potency, practicality and cost-effectiveness of copper-coated surfaces are needed. Therefore, a differential approach for risks versus benefits analysis in healthcare settings is needed.

Currently, the global market for AMCs is estimated worth of $1.5 billion, while the global AMC demand is prognosed to reach $2.9 billion in 2018 [27]. In spite of all beneficial effects of AMCs, their introduction in healthcare settings, together with different methods for cleaning, may cause significant changes in the emission of toxic agents into the environment. Active ingredients released from coatings are likely to slowly enter the ecosystem leading to exposure and possible adverse effects in human, livestock and microbiota. In addition, the slow infusion of antimicrobial nano-ingredients may induce AMR that differs from current antibiotic driven mechanisms [28]. The widespread introduction of such coatings therefore needs to be subjected to risk-benefit analysis. Already in 2009, the Scientific Committee on Emerging and Newly Identified Health Risks (SCENIHR) recommended in its opinion on AMR “prudent use of antimicrobials”, “reduction of the overall use of antimicrobials in a balanced way in all areas” highlighting an urgent need for assessment of major AMR contributors [29].

In contrast to the surveillance on the use and production volumes of antibiotics for human and animal health care, biocides used in AMCs are not regularly monitored. Therefore, the assessment of AMR generic risk of biocides used in AMCs requires following issues to be solved:

(i) Information on production and use volumes of particular biocides in current use;
(ii) Epidemiological data indicating public health relevance of AMR;
(iii) Data on the environmental stability and fate of individual products;
- (iv) Dose-response relationship and of the threshold triggering the emergence of AMR;
- (v) Generation of standards for the testing and surveillance of AMR at the international level.

Through its Cooperation in Science and Technology programme (COST), the European Commission has recently funded a four-year initiative (2016–2020) to establish a network of stakeholders involved in development, regulation, and use of novel AMCs for the prevention of HCAI. The network AMiCI (AntiMicrobial Coating Innovations) currently comprises participants of more than 60 universities, knowledge institutes, and companies across 29 European countries representing the most comprehensive cluster to target use of AMCs in healthcare settings on surfaces in the environment of patients [30].

The current opinion paper aims to guide the reader through different aspects of quality and safety aspects of AMCs use and application on frequently-touched surfaces in healthcare settings. We particularly focus on potential adverse effects such as induction of AMR and/or ecotoxicological effects, needed for the risk-benefit evaluation of application of these novel coatings. This paper was inspired by facilitated discussions (flip-chart sessions) with experts from universities and research institutes as well as from non-academic-scientific institutions (producers, suppliers, advisors, hospitals, etc.) during kick-off meeting of COST Action CA15114 AMiCI in Heerlen Netherlands, 17 November 2016.

## 2. Materials and Methods

AMiCI consortium members (*n* = 85) were invited to facilitated discussions (flip-chart sessions) during the kick-off meeting of COST Action CA15114 AMiCI in Heerlen, The Netherlands and asked to familiarize themselves with the questions shared with them two weeks before the forum discussions. In total, 75 AMiCI members from 24 European countries shared their opinions in the forum discussions that were held in Heerlen. Analysis of the professional affiliation of AMiCI participants who attended the flip-chart discussions showed that ~90% of the participants were from universities and research institutes and ~10% were other stakeholders such as AMC producers, suppliers, advisors, hospital microbiologists etc.

The questions for the flip-chart sessions were based on the topic of COST AMiCI working group 3 (WG3) focusing on (eco)toxicological risks and the possible induction of AMR resulting from the use of AMCs in the healthcare settings. Altogether, four main topics were addressed and formulated as questions:

(i) What are the possible (eco)toxicological risks related to application of antimicrobial materials in healthcare settings?
(ii) What are the possible risks related to potential development of antimicrobial resistance?
(iii) Could these risks be addressed at the level of ‘safe-by-design’ of antimicrobial coatings?
(iv) Adverse effects/risk-benefit analyses: who should be involved in the process?

The discussion was preceded by a plenary lecture on the theme given by Anne Kahru, leader of the WG3 in AMiCI COST Action. For the flip-chart discussions, the participants were divided into four subgroups and discussion of each question lasted for approximately thirty minutes facilitated by one or two key members of AMiCI WG3 (authors of this paper). First, a short introduction was provided by the facilitator as well as the posing the additional questions. The answers and comments were written on flip-chart(s). After thirty minutes, the group moved on to the next question. Thus, all participants shared their opinions on all four questions. After the meeting, the facilitators summarized the results of the discussion and this information was used to guide the writing of this paper. Structure-wise, we follow the above described sub-topics (four questions) and back-up each of them with information originating from the scientific literature and relevant reports.

## 3. Results

Herewith, each of the four individual questions posed is considered separately combining the results of flip-chart discussions with analysis of the data from the relevant scientific literature. As the work of AMiCI network focuses on the use of antimicrobial materials on surfaces near patients (especially on frequently-touched surfaces), the results and studies on AMC enabled medical devices are not discussed and presented in this paper.

### 3.1. Question 1: What Are the Possible (Eco)toxicological Risks Related to Application of Antimicrobial Materials in Healthcare Settings?

During the flip-chart sessions it was admitted that most of the participants had not the appropriate background knowledge to address the topic of environmental issues connected with AMCs. Therefore, the view on this topic described here is the summary of the literature data and experience of the authors rather than general opinion of the AMICI consortium. We will address and comment here the main topics that resulted from the discussions during the flip-chart discussions.

#### 3.1.1. AMCs: Classification and Mechanism(s) of Action

**Figure 2:**
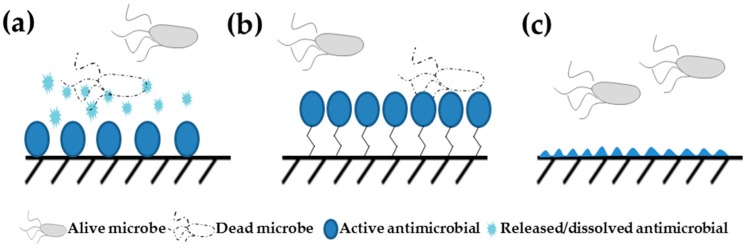
Different types of AMCs: (**a**) antibacterial agent release-based coatings; (**b**) contact killing based surfaces; and (**c**) anti-adhesion surface with specifically designed surface topography. NMs are mostly applied in release-based or contact killing surfaces.

In a broad view, AMCs may be classified (Figure 2) [2] as:

- Coatings that release the active substance; these are the oldest and most commonly used coatings prepared by simple impregnation, soaking or coating of a porous material with antibacterial compound;
- Coatings that have the active substance covalently anchored to the surface;
- Anti-adhesion surfaces which are specifically designed surface topographies that repel microbes or decrease their surface attachment.

The majority of AMCs are based on the release of the active biocidal agent from the surface (Figure 2a). For example, the active antimicrobial agent in nanosilver-based coatings is silver ion released from nano-enabled surface [31]. In most of the current silver-based AMCs, silver NPs are either deposited directly on the device or present inside polymeric surface coatings. For these applications slower release of active agents has been even considered as a disadvantage due to the possibility of developing resistant microbes. However, usually longer shelf life of the surfaces is desired.

Contact killing of microbes utilizes the interaction(s) of microbial cells with the surface and subsequent inhibition (Figure 2b). The interaction between microbial cells and the surface may be accidental or directed and the inhibition of microbes can be due to the release of an antimicrobial agent in the close vicinity to the surface, or due to e.g., surface topography. In addition to bacterial killing, reduction of bacterial number on certain surfaces is also possible to achieve by using anti-adhesive, i.e., repelling surfaces (Figure 2c). In general, bacterial adhesion to a surface depends on surface topography, hydrophilicity/hydrophobicity and surface molecules. For example, a perfectly smooth surface will be less likely populated by bacteria than a rough surface, where more adhesive force can be generated by a microorganism per surface area [32,33]. Also, hydrophilic surfaces will be less quickly populated by bacterial cells than hydrophobic ones due to the presence of hydrophobic patches on the outer surfaces of most microorganisms. In real conditions however, the adhesion of microorganisms almost always depends on formation of a protein layer on AMC and the presence of adhesion sites on that formed protein layer [32].

In order to achieve efficacy but also safety, the design of AMCs, that often incorporate a biocidal chemical or material, is faced with several challenges. These challenges are discussed below.

#### 3.1.2. Biocidal Chemicals Used in Antimicrobial Coatings Are Inherently Toxic

Biocidal products are necessary to avoid the proliferation of organisms that are harmful to human and/or animal health or may damage the natural or man-made materials. As a rule, the biocidal products are intrinsically toxic, i.e., show harmful effects towards different types of cells/organisms due to the inhibition of the proliferation of target organisms (such as pathogenic bacteria or biofilms of microorganisms or algae) [34]. In addition, these biocidal products can be harmful to humans, animals and to the environment at large [35].

One group of biocidal materials that are inherently toxic to most biota, starting from microorganisms and ending with vertebrates are metal-containing biocides, most notorious of them being Ag, Cu and Zn. The soluble salts of these metals are very toxic to the most of aquatic organisms, while released metal ions (Ag^+^, Cu^2+^, Zn^2+^) are claimed to be one of the main reasons of the toxicity of metal-based NPs that are prone to solubilization in the aquatic environment or moist conditions [36]. In addition to being toxic to aquatic organisms, the extensive use of these metallic biocides may contribute to the development of AMR (see below).

The meta-analysis of the scientific literature on toxicity of NPs of Ag, CuO and ZnO—(nano)materials often used as antimicrobials—showed that these compounds in the conditions they were tested proved remarkably more toxic to ecotoxicological test organisms such as aquatic crustaceans and phytoplankton (algae) than to bacteria (Table 1). This information shows that certain antimicrobial compounds may be more harmful to aquatic organisms than to microbes, necessitating the analysis of toxic impact of environmental waste flows of these compounds. It is important to note that in Table 1 (i) the toxicity endpoint in the case of bacteria is minimal inhibitory concentration (MIC) which means no bacterial growth at this concentration of chemical, and not the half-effective concentration (EC50) that is used as a toxicity endpoint for ecotoxicological test organism, i.e., exposure concentration at which 50% of organisms are alive and (ii) as the toxicity of the above mentioned compounds is driven by shed metal ions, the speciation dictates the final toxicity and heavily depends on metal and test media composition [37]. Speciation is also an important parameter in the analysis of the environmental effects (product life cycle) of metallic pollutants, including metallic biocides used in AMCs as discussed below in this paper.

#### 3.1.3. Relevant Regulations Involved in Europe

The compounds containing silver, copper and zinc are covered within EU by the Biocidal Product Regulation (BPR, Regulation (EU) 528/2012) and by the Regulation (EC) No 1907/2006 of the European Parliament and of the Council on the Registration, Evaluation, Authorization and Restriction of Chemicals (REACH).

#### 3.1.4. Risks Arising Due to the Use of Antimicrobial Coatings in Healthcare Settings: Silver Nanoparticles as a Model Compound

According to meta-analysis of the scientific literature, silver is prevailing metal used in AMCs (30% of papers; Figure 1). Similar tendency can be seen among nano-enabled consumer products: Nanotechnology Consumer Products Inventory created by Woodrow Wilson International Center for Scholars and the Project on Emerging Nanotechnologies (latest revision released in October 2013) [38] shows that in 442 out of 1814 (24%) consumer and medical products silver is the most frequently used NM. TiO_2_ is included in 92 products, SiO_2_ in 43, gold in 25 and copper in 10 products [38].

Silver is also the most frequent NM in Danish Nanodatabase [39] (384 products or 15.9%) launched in 2012 by the Danish Consumer Council and Ecological Council and the Technical University of Denmark’s Department of Environmental Engineering. In addition, there is already considerable amount of scientific information available on potential harmful effects of AgNPs to humans and environment. We will briefly discuss the most relevant and recent reports here.

• *Nanosilver dossier by project ‘Nanotrust’ (2010)*

A dossier concerning AgNPs [40] was composed by scientists of Institute of Technology Assessment of the Austrian Academy of Sciences as result of the project ’NanoTrust’. The dossier addressed following concerns of adverse effects of use of AgNPs: (i) the development of silver-resistant bacteria may be induced by release of subtoxic levels of silver ions; (ii) human skin microflora may become compromised after use of AgNP-enabled cosmetics; (iii) if discharged into wastewater, silver may accumulate and elevated silver concentrations may adversely affect aquatic environments and microbial communities in wastewater treatment plants (WWTP) and soils. 

• *SCENIRH Report on nanosilver (2014)*

Chronologically the next comprehensive study on AgNPs was conducted by SCENIHR (Scientific Committee on Emerging and Newly Identified Health Risks) on request of the EC and approved by SCENIHR in June 2014 [41]. This study aimed to evaluate whether the use of AgNPs, in particular in medical care or in consumer products, could result in harmful effects, e.g., the development of AMR. The authors concluded that the hazard associated with the exposure to AgNPs can be also related to increase of bacterial resistance to silver but also to other antimicrobial compounds, but there is currently a serious gap of knowledge in this area [42]. The report is a document of 103 pages and one of the conclusions was that *...*’*A detailed risk assessment of nanosilver has not been performed since too little information is available...*’.

• *OECD WPMN Report on exposure to nanosilver (2016)*

The most recent extensive report was published by OECD Working Party on Manufactured Nanomaterials (WPMN) that addressed exposure to AgNPs. It is important to note the exposure is very important category in risk assessment: if there is no exposure to a toxicant, there is no risk. As the report was published in 2016, it describes ’state-of-the-art’ with purpose to identify existing data gaps regarding exposure assessment of AgNPs and to make recommendations on how to address these data gaps. One of the recommendations reached by the experts was the need for (i) collection of more detailed information on releases of AgNPs from products or applications over the entire life cycle into the different environmental compartments (e.g., WWTP, surface water, sediments, soils); (ii) development of appropriate methods and models to specifically estimate the environmental exposure of AgNPs; and (iii) accounting for the sum of environmental exposure of AgNPs from different sources [43].

• *Additional pertinent information on silver nanoparticles from the literature*

There is already considerable information accumulated concerning environmental hazard of some types of nanosilver-enabled products. For example, up to 10% of the silver of the nano-enabled textiles can be washed out and enter the environment via WWTPs [42]. In the WWTP up to 99% of the AgNPs can be removed via sewage sludge via transformation into water-insoluble silver chloride and -sulfide [44]. As sewage sludge is often used in agriculture, the soil may be the main deposition site for AgNPs [45]. Fortunately, due to speciation the mobility of silver in the soils is very low [41]. The other important route for deposition of nano-enabled compounds/products is landfilling: worldwide 60 to 86% of the most commonly used engineered NMs end up in landfills [44]. In simulated landfill conditions, for example, the residual silver from (nano)silver-enabled textiles was still continuing to leach [46]. Thus, due to the complex speciation, AgNPs in the environment will be transformed via agglomeration, dissolution, speciation, sulfidation, sorption to sediment and soil particulate matter [42]. However, concerning the hazard evaluation of AgNPs, it is generally agreed that fate of AgNPs remains largely unknown, even in the aquatic environment (that is a ’simple’ environmental compartment compared with complex matrices such as sediments and soil—authors’s remark) [47]. 

During flip-chart discussion on this sub-topic, we agreed with the above described reports and publications prepared by top-specialists in the field and cited here: too little information is currently available to conduct a detailed risk assessment even for AgNPs. The data gaps are even more severe for other (nano)antimicrobials that have remarkably less available information needed for the procedure of risk assessment over the entire life cycle into the different environmental compartments.

### 3.2. Question 2: What Are the Possible Risks of Antimicrobial Coatings Related to the Potential Development of Antimicrobial Resistance?

The AMiCI flip-chart discussions resulted in mutual agreement on possible AMR development connected to AMCs. It was pointed out that horizontal gene transfer of AMR to pathogenic ones or to environmental bacteria is a realistic scenario. As corresponding scientific data are insufficient, prudent use of AMCs, i.e., only in limited areas was advised. The influence of contact time and concentration, cleaning procedures and hospital wastewater management were the aspects pointed out as important precautions to avoid resistance development and possible transfer to environmental microbes. Also, the concern of laboratory scale test results to represent the complex real life environmental conditions (e.g., hospital, sewage sludge, sediments, soils) was raised. The above-mentioned topics will be discussed below in the context of pertinent scientific literature.

#### 3.2.1. Antimicrobial Resistance as a Global Problem

Nowadays AMR among pathogenic and non-pathogenic microorganisms has become a serious global threat. In fact, natural antibacterial agents have influenced microbial ecosystems during billions of years and development of different resistance mechanisms was essential for microbial survival [48]. Although humans have empirically used different natural antimicrobial substances including silver and copper from early history, the widespread use of antimicrobials has speeded up in parallel to the start of commercial production of various antimicrobials such as arsphenamines at the beginning of the 19th century [49,50]. Ever since antimicrobials have saved uncounted number of lives but as negative side antimicrobial resistance has followed the introduction of every new compound [49].

#### 3.2.2. Antimicrobial Coatings as Potential Inducers of Resistant Microbes

**Figure 3:**
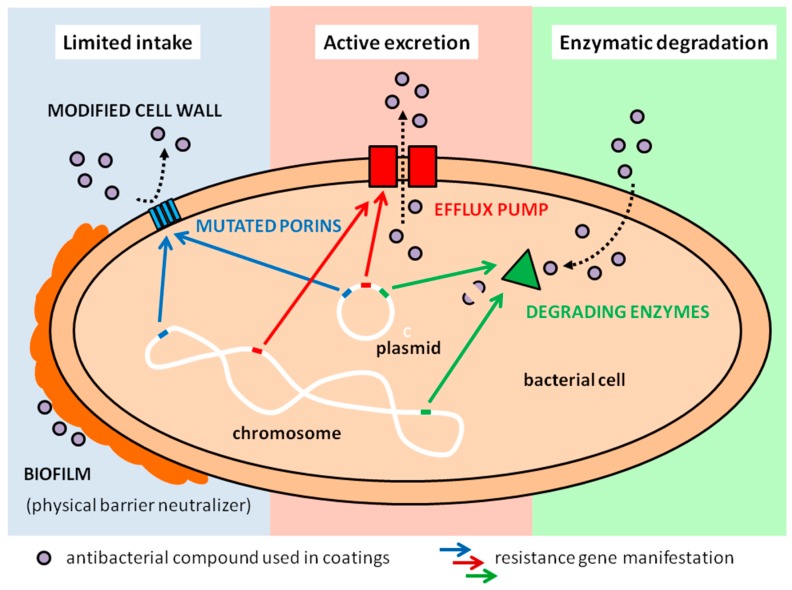
The main proposed mechanisms of AMR against antimicrobial compounds; a simplified scheme.

The growing use of AMCs in recent years has increased concerns about appearance of resistant microbes. Active components of AMCs (e.g., silver, copper, zinc, titanium dioxide, and their nanosize forms as well as more traditional antiseptics like quaternary ammonium and chlorhexidine) possess broad spectrum of antimicrobial activity as they target multiple sites on and within the microbial cell. Therefore, their antimicrobial action is different from antibiotics that are usually directed against specific bacterial structures [51,52]. There are three main proposed mechanisms of AMR against antimicrobial compounds used in coatings: (i) active excretion; (ii) limited intake and (iii) enzymatic transformation of the antimicrobial agent (Figure 3). These mechanisms may occur concurrently. Among them the efflux pumps localized in the cytoplasmic membrane of the cells pumping out various substances are the most commonly described [53,54]. This is non-specific resistance mechanism and often occurs at the same time with the antibiotic efflux leading to multi-resistance [54]. Additionally, described resistance mechanisms involve altered biocide permeability such as the lack of specific porins in the outer membrane of bacteria necessary for passive diffusion of molecules or changes in outer membrane ultrastructure and surface hydrophobicity [55,56]. Formation of bacterial biofilms, i.e., bacterial aggregates instead of single planktonic bacteria can also limit the diffusion, interact or neutralize the effectiveness of AMCs [57]. Also, enzymatic degradation of antimicrobial agents from toxic to less toxic compounds has been detected [58]*.*

Several resistance genes encoding enzyme synthesis responsible for resistance towards metallic as well as non-metallic compounds used in AMCs have been described [59,60]. The resistance genes may occur in bacterial chromosome or in plasmids and can be transmitted to other microbes via horizontal gene transfer similarly with antibiotic resistance genes [60]. For example, resistance genes for silver that have already been found in hospital environment [61,62] are located in mobile genetic elements easily transferable to other bacteria e.g., in (hospital) sewage systems [63]. Expression of biocide resistance genes has been associated with concurrent AMR [60,64]. Regrettably, common knowledge on the appearance of resistance towards AMCs is still very limited with only a few reports being published.

#### 3.2.3. Contribution of Antimicrobial Coatings to the Development of Antimicrobial Resistance: Data-Gaps

One of the main reasons behind the wide use of biocidal NMs in antimicrobial applications is their potency in replacing conventional antibiotics, which is impeded by frequently occurring AMR. As recently stated by the World Health Organization [65] “*AMR is a complex problem driven by many interconnected factors so single, isolated interventions have little impact and coordinated actions are required.*” Long duration exposure to antibiotics in sub-inhibitory concentrations has shown to lead to AMR [66]. However, knowledge on the influence of low dosages of active components used in AMCs to the development of AMR is lacking. The issue is especially intriguing in case of AMCs containing NPs where the concentrations of nano-antimicrobial agents are several times lower than in conventionally used ionic compounds.

Also, no reliable information is available on the continuous influence of ultra-low concentrations of antimicrobial agents as NPs are reaching human tissues and microbes of normal microbiome. Most of our scientific knowledge on the influence of AMCs to microbial resistance is laboratory-based with limited variables while in the real life, especially in hospitals, more complex conditions occur: due to widespread use of antibiotics and disinfectants the selection of the multidrug resistant bacteria emerges. In hospital wastewaters, pathogenic and non-pathogenic bacteria with different resistance genes and gene complexes, residues of antibiotics, disinfectants, cleaning agents and different nutritional components meet and uncontrolled biological processes take place. The influence of antimicrobial NPs in these processes is suspected, but not scientifically proven [67]. Hospital wastewater has been shown to contain different multidrug resistant bacteria and untreated effluents play important role in the spread of the resistance among the bacteria to the environment outside the hospitals [68].

The cleaning of AMCs in hospitals is also an important factor to consider. Continuous mechanical and chemical influence of cleaning agents may reduce the activity of AMCs to sub-inhibitory concentrations and lead to generation of resistance. Antagonistic interaction between some liquid disinfectants has been described [69]. Chen et al. [70] have described antagonism between nano-TiO_2_ and copper ions. Still, not enough information is available about interactions between cleaning agents and AMCs for everyday practice. Therefore, the generation of science based guidelines for proper cleaning practices are needed.

**Figure 4:**
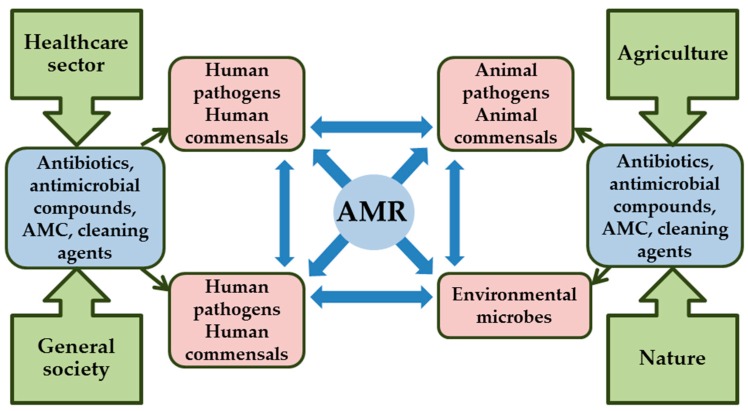
Conceptual model for the development of antimicrobial resistance (AMR) in response to antibiotics, antimicrobial compounds, antimicrobial coatings (AMC) and cleaning agents, and transfer of AMR between different microbial populations: human, animal and environmental.

The potential development of AMR due to biocidal formulations in AMCs may cause hazard to different groups of organisms and humans as well as the environment at large. In order to understand and minimize the risks of developing AMR to antimicrobials or AMCs, it is vital to consider all biological and environmental routes (Figure 4) which enable these bacteria and their genes spreading between different organisms as reviewed by da Costa et al. [71].

#### 3.2.4. Development of Antimicrobial Resistance Due to Antimicrobial Coatings: Potential Risks at Different Levels and over the Coatings’ Life Cycle

On one hand, AMR bacteria developed in hospitals may spread to environment through sewage water or sludge. On the other hand, AMR bacteria developed due to veterinary medicine practices create a selective pressure for the emergence of resistance among bacteria present in animals; animal pathogens, human pathogens that have animal reservoirs, and commensal bacteria. Examples of environmental hot spots for possibly high-level of horizontal gene transfer and antibiotic resistance include aquatic environments that are affected by pharmaceutical industry effluents, aquaculture, or sewage discharges, and terrestrial environments that are affected by the deposition of biosolids or animal manures. The described ecological framework provides an essential perspective to evaluate antimicrobial use risks and policies, because it contains the root causes of these problems rather than merely their consequences [71].

### 3.3. Question 3: Could the Ecotoxicological Risks and Risks Related to Potential Development of Antimicrobial Resistance Arising from the Application of Antimicrobial Materials in Healthcare Settings Be Addressed at the Level of ‘Safe-By-Design’?

This question aimed to gather AMiCI experience with current methodology, guidelines and regulatory requirements to manage AMCs development at the Safe-by-Design (SbD) level. During the flip-chart session, participants recognized difficulties related to experience and knowledge concerning safety assessment of AMCs. The consensus of AMiCI workshop participants was that Quality, Efficacy and Safety (QES) management of AMCs could best be achieved by applying SbD approach at points of antibiotic manufacturing and use. Although battle for successful SbD implementation is ongoing, greater support from funding and regulatory agencies is needed due to major uncertainty problems that NMs pose.

As already stated earlier, AMR and (eco)toxicology were not the areas of core expertise of the majority of participants. Hence, authors of the paper piloted whole discussion toward final response and opinion on this issue. 

#### 3.3.1. Safe-By-Design Approach in Antimicrobial Coatings Use and Development

SbD is a well-accepted approach, developed within the European research projects NANoREG, ProSafe and NanoReg2, for timely identification of risks related to the industrial innovation processes and value chain of nanomaterials and nanoproducts. It is designed to ensure safety for three different, but interrelated communities—the workplace, consumers and the environment. The basis of SbD approach is summarized in Table 2.

**Figure 5:**
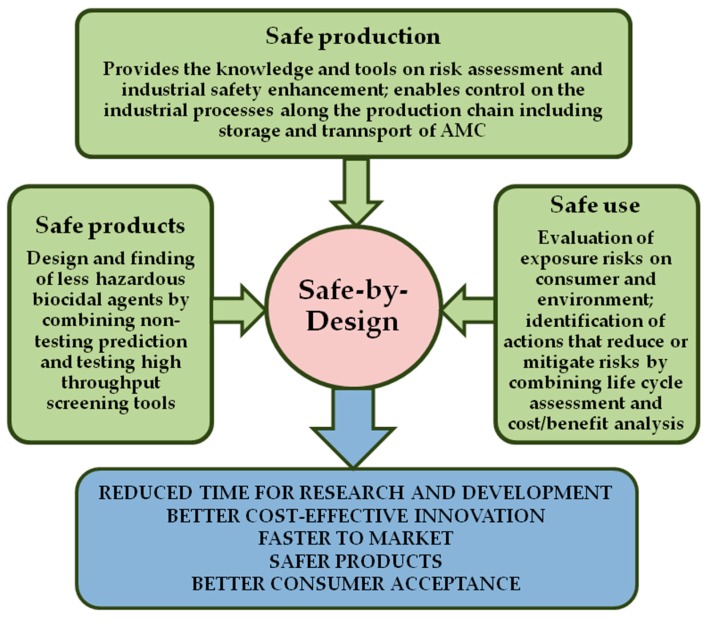
SbD approach in safe production and use of AMCs.

The SbD concept becomes central to numerous nanotechnological and nanosafety projects that are finished, ongoing or planned in the EU. A common SbD approach, as presented in Figure 5, is characterised by,

- Convenience of integration into existing industrial innovation processes;
- Early and easier identification of uncertainties and risks;
- Reduction of uncertainties and risk;
- Timely recycling or termination of projects with unacceptable risks;
- Decrease of a number of unforeseen events during the development process and market introduction;
- Preparedness for current and future regulatory requirements;
- Balanced safety, functionality and costs of final product;
- Improved design of products and better business models.

#### 3.3.2. Quality, Efficacy and Safety Assessments of Antimicrobial Coatings

In spite of the considerable amount of published data on AMCs development and use, comprehensive knowledge on the interactions of AMCs with biological and environmental systems is still lacking. Main reason for uncertainties on safe and efficient AMCs application may be attributed to challenges of experimentation with nanomaterials [72]. During manufacturing, application and final disposal, nanoparticles may undergo different bio-physico-chemical changes. The nano-specific behaviour is especially relevant for exposure, absorption, distribution, accumulation, and toxicity effects [73]. Most of the methods used for QES assessment have been designed and standardized for traditional chemicals. However, these methods can not necessarily be used for NMs. Unique physicochemical properties of NPs, like high adsorption capacities, optical properties, increased catalytic activities, often interfere with the readouts of many in vitro toxicity assays, leading to the false interpretation of results [74]. Examples of such interferences include: increased adsorption capacity due to the high surface area; effects on fluorescence or visible light absorption detection due to different optical properties; increased catalytic activity due to enhanced surface energy [75]. In addition, careful characterisation of NMs applying a multi-method approach should be performed to determine quality, efficacy and safety of a substance produced by nanotechnology. An exhaustive characterization of NPs is often time consuming, expensive and complex. Such approach requires well-equipped laboratories with all the necessary facilities and competences. Thus, current industrial innovation processes and risk management for nanomaterials have to be enhanced by the SbD concept which is designed towards early and easier identification of uncertainties and risks related to the production and use of NPs.

QES assessments of AMCs would be able to provide reliable measures for reduction, or even elimination of these uncertainties and risks during an innovation project. This assessment should be amended by discussion with all relevant stakeholders, which requires extensive risk communication and could form part of the multicriteria decision analysis. During this process, there are several research gaps that need to be addressed. In particular, specific attention should be paid to hot spots where prevalence of biocides, co-selecting agents, bacteria carrying resistance determinants on mobile genetic elements, and favourable conditions for bacterial growth and activity develops at the same time.

From the perspective of SbD concept, QES assessment of AMCs should encompass: (i) assays for determination of minimum selective concentrations [76] that are validated in different environmental matrices with isogenic pairs of the model organism inoculated into the matrix of choice applying sub-inhibitory concentrations; (ii) assays for identification of environmental hot spots where a high-level of horizontal gene transfer and antibiotic resistance develop like aquatic environments affected by effluents from pharmaceutical industry, aquaculture, or sewage, and also terrestrial environments affected by the deposition of biosolids or animal manure; (iii) screening procedures for novel resistance determinants to ensure that existing resistance determinants are not prevalent in environmental compartments; (iv) dose–response data that address people of various life-stages; (v) strategy risks ranking based on exposure assessment modelling [77].

#### 3.3.3. The Use of Safe-by-Design Principle in the Development of Antimicrobial Coatings

As described in previous chapters, SbD concept encourages elimination of health and safety risks already during product construction and development. By considering the functionality and toxicity of AMCs in an integrated way as suggested in SbD approach, further development process can offer smart innovation in making AMCs fit for succesful economy. During design stage in AMCs development, several different strategies can be considered.

Currently, the majority of AMCs are based on the release of the active chemical from the surface (Figure 2a) as discussed above. The typical profiles of release of active agents from nano-enabled coatings follow first- or second-order kinetics, i.e., exhibit an initial burst release which is followed by a decreasing tail usually ranging from hours to some days [2]. This usually results from weak bonding of the active agents, e.g., NPs to the surface, but could be overcome by using various methods to incorporate the NPs to the coatings more tightly. Such an attachment has been reported for AgNPs to –SH groups on glass surfaces [78]. The authors claimed no release of NPs from surface, while ca. 15% of soluble silver was released over 19 days [78]. Other means to decrease the release of active substances from antibacterial surfaces include a wide variety of encapsulation materials and deposition strategies. The most often used carriers include poly(methacrylic acid), polyacrylic acid, poly(lactic-co-glycolic acid), polyurethane, hyaluronic acid, chitosan and hydroxyapatite [79,80]. Another popular strategy to control the release of antibacterial agents from coatings is the use of polyelectrolyte multilayers that are nanostructured polymeric systems trapping antibacterial agents [81]. A recent study showed that using a polyacid core in polyelectrolyte multilayers could lead to significant sustained release of the antimicrobial agents: while releasing physiologically-relevant drug concentrations, the coating remained stable and functional over 14 months [82].

In general, the strategies to reduce and control the release of active agents from AMCs can be divided into four main categories: (i) passive strategies that control the antimicrobial release by tuning the carrier matrix to a suitable size (e.g., controlling the size of pores into which the antibacterial is loaded [83,84]); (ii) passive strategies to control antimicrobial release by adding a thin polymer layer, where the thickness, hydrophobicity and the degree of crosslinking of the polymer layer have been shown to be the main factors affecting the release kinetics [85,86]; (iii) stimuli-responsive antimicrobials that contain polymers which shrink, swell or bend [87,88]; (iv) active control over the release of antimicrobial agent by the microorganism itself, e.g., pH responsive polymers that change their conformation under acidic conditions, after the release of various metabolic intermediates by bacteria [89] or agents immobilised onto the NPs surface using pH sensitive amide bonds [90].

In addition to controlling the release of biocidal agents from AMCs, contact killing strategies can be applied to increase the safety by design of AMCs (Figure 2b). Perhaps one of the best known examples of contact killing of bacteria on surfaces is a ’sharkskin’ [91]. This is a micropatterned surface which is made of polydimethylsiloxane elastomer and that initially proved antifouling towards green algae and later also for bacteria [92]. Release of biocidal agents can be also combined with contact killing. For example, Li et al. [93] combined bilayers of AgNPs with immobilized quaternary ammonium compounds and polymers using layer-by-layer deposition. The results showed that during first few days, the antimicrobial effect of such coatings was driven by release of silver while the quaternary ammonium compounds retained significant contact-killing activity at later timepoints [93]. The 3rd appoach in creating AMCs is design of special surface topography that also can be used as a SbD strategy for AMCs (Figure 2c). Differently from contact killing where low quantitites of biocidal material can be released, no biocide is released from surface topography controlled AMCs. As an example, Kim et al. [94] prepared porous alumina surfaces with different pore sizes that depending on the pore size exhibited different killing efficacy for bacterial cells. In addition to specially designed surface topography, bacteria-repelling surfaces have been shown as an efficient biocide-free SbD strategy to design AMCs. Such repelling surfaces may be remarkably smooth or functionalized e.g., with PEG chains [95].

An ideal SbD strategy for AMCs design could be specific targeting of certain microbes with negligible effect to other microbes and environmental organisms. It must be, however, noted that due to the broad range of effects of AMCs and their similar toxicity pathways in different organisms, such a selectivity is difficult to obtain. Therefore, such organism-specific AMCs can only be achieved through the immobilization and microbe-specific release of the antimicrobial agent/NM. Most of succesful strategies for the discrimination between different microbes are based on bonding of the antibacterial compound to the substrate by an enzymatically cleavable bond [96,97,98].

As discussed above, various ways could be used to implement SbD concept into current industrial innovation processes in order to address the safety in the workplace, for the consumers and the environment. 

### 3.4. Question 4: Adverse Effects/Risk-Benefit Analyses of AMCs: Who Should Be Involved in the Process?

Beyond the level of risk-benefit evaluation along the production-usage-disposal chain, the final analysis concerning all the advantages and disadvantages is a very challenging task. It has been shown that the risk-benefit analysis of nanobiocides (e.g., AgNPs) can lead to the negotiated risk [99] and therefore all the concerned stakeholders should be involved already at the beginning of the risk-benefit analysis of AMCs.

During flip-chart sessions of the AMiCI meeting (see above) the following list of the institutions and organizations who should be involved in the AMCs risk-benefit analysis was drafted:

(i) Producers, distributors and suppliers;
(ii) End-users (hospitals, medical advisors, patients’ associations);
(iii) Research institutions (research and development, know-how and expertise);
(iv) Regulatory and standardization agencies (standards, reference materials, threshold values);
(v) Environmental and health agencies (monitoring and safety assessment along the life cycle, exposure at the workplace, epidemiological data);
(vi) Media, mass and social communication (dissemination of knowledge on AMCs innovation and potential risks to a wider audience avoiding negative public perception caused by the lack of information).

Participants of the flip-chart sessions stressed that a multi-disciplinary approach involving scientists, medical doctors, producers and regulatory organizations is required in the decision-making process to put new AMCs on the market and to adopt them in the healthcare settings.

Although a huge amount of lab work, involving chemists, physicists, material scientists and biologists, has been made to identify new efficient AMCs, this is only the first step towards a realistic beneficial application of AMCs in the healthcare settings. The following critical step is the technology scale-up where scientific, technological and economic costs and benefits should be carefully weighed. Focusing on the risks, this represents the moment when the impacts toward the environment and human health must be evaluated considering the whole material’s life cycle. All these pre-commercial studies are needed to guarantee the safety in parallel with the increased antibacterial efficacy of the new AMCs. The environment and humans in fact may be exposed to AMCs during production, transportation, usage and disposal phases, and especially when the materials used fall into the category of the emerging contaminants—as the NMs are—special attention should be paid. Environmental scientists, physicists, chemists, toxicologists and occupational medical doctors are expected to fulfill the knowledge gaps still existing on the possibility that some adverse health effects might be really associated to relevant exposure to the new AMCs or their by-products. Searching of ISI Web of Science database (22 January 2017) using separately the keyword antimicrobial coating* or combining that with ‘risk’ or ‘benefit’ or ‘risk and benefit’ resulted in 3377, 173, 72 and 14 articles, respectively, and most of the risk-benefit articles concerned AgNPs-coated catheters, showing that there are still extensive data gaps for evaluating the risk and benefit aspects of AMCs and referring to the need of more comprehensive studies.

In order to obtain reliable information on the AMCs efficacy and to provide assurance for the end users, standardised protocols are needed [100] as was also pointed out during COST AMiCI meeting discussions emphasizing that the lack of standardized tests and threshold values for AMCs (e.g., release concentrations of biocidal compounds etc.) complicates the risk-benefit analysis. Therefore, it is important to involve also regulatory and policy-making organizations in the analysis/discussion. Although the European Committee for Standardisation aims to produce current and future European disinfectant testing standards (CEN/TC216) and the OECD Working Party on Nanomaterials published in 2012 a ‘Guidance on sample preparation and dosimetry for the safety testing of manufactured nanomaterials’ [101], there are still no harmonized standard protocols for NM and biocides testing [65,77]. Testing methodology usually ranges from basic preliminary suspension tests to more complex protocols that simulate conditions in practice. The design of robust and reproducible test protocols for efficacy and safety evaluation of AMCs is quite complex due to the number of factors that need to be controlled, such as microbial test strains, preparation of inoculum, detection and count of survivors, quenching antimicrobial activity, physical parameters. In addition, there is no standardised testing strategy for assessing both biocide and AMR in bacteria. Instead, environmental and clinical isolates are usually tested for their susceptibility to biocides and antibiotics using separate protocols.

One aspect stressed by the participants of flip-chart sessions was the involvement of media to disseminate the knowledge on AMCs innovation and potential risks to a wider audience. Media involvement is very useful especially if the risk-benefit analysis showed that the risk should be considered as negotiated risk to avoid the negative public perception. Scientists as the highest trusted group in the society should be involved in the risk and benefit communication [102].

In conclusion, the participants of the discussions proposed that the AMCs risk-benefit analysis should involve scientists (chemists, physicists, material scientists, microbiologist, toxicologists, etc.), producers, end-users (hospitals, healthcare institutions), governmental and non-governmental organizations (e.g., patient’s organizations, environmental and chemical agencies) and also media. Involvement of various stakeholders is useful for the meaningful risk-benefit analysis and for the better regulation (e.g., standards, guidelines) [103]. Although most of the participants of the flip-chart sessions were from the research institutions (~90%), quite a lot of the suggestions were made by the representative of producers, pointing that the producers need professional support and information for the AMCs development, risk assessment and authorization.

## 4. Different Aspects of Risk-Benefit Analysis of Application of AMC in Healthcare Setting

Critical questions have been asked whether it is wise to dispatch this powerful antibacterial weapon (i.e., AMCs) for regular use or rather hold back until well-designed clinical trials show the real benefits of such coatings. Indeed, in 2010 there was even a lawsuit concerning nanosilver-based textiles against Swiss company HeiQ to which US EPA granted registration in 2011 after having made incorrect assumptions [104]. On the other hand, although in vitro adverse effects have been seen, systemic acute in vivo toxicity of AgNPs is relatively low. For example, in a study of patient(s) with severe organ argyrosis [105] demonstrated numerous diffuse areas of silver deposits but no nephrotoxicity. Similarly to AgNPs, no clear in vivo toxicity has been related to any other NM. Although current reports indicate relatively low nanotoxicity in vivo, it remains to be determined if NPs used in AMCs are safe for patients in the long run. 

As discussed above, currently there is not enough information for conducting environmental risk assessment for application of AMCs in healthcare setting. Concerning the potential development of AMR, most of the active compounds in AMCs have been used in antimicrobial purposes through long time period both in agriculture and in healthcare but no widespread resistance has emerged (although resistance genes to some of the active compounds in AMCs already exist). The potential development of AMR in response to the widespread use of AMCs may lead to risks in healthcare as well as in agriculture and environment in general. Still, as not enough scientific data about AMR connected to AMCs, especially nanocoatings is available by now, lessons learned from antibiotic resistance must not be forgotten. Our task should be the reasonable and controlled use of AMCs not only in the hospitals but also in the community. However, in order to minimize the risks, AMCs should be used in restricted circumstances. In hospitals, the use of AMCs should be considered in hot spots with frequent hand contact (e.g., knobs, switches, rails) but only in high risk areas with immune-compromised patients/hosts. Using AMCs in the community ought to be limited to selected areas with possible high infection transmission risk such as schools, prisons, athletic and military settings but merely in case of proven infectious hazard.

Disinfection of hospital wastewater consisting of different multidrug resistant bacteria could be one of the means to reduce the spread of the resistance among the bacteria outside the hospitals. Still, not enough information is available about interaction between cleaning agents and AMCs for everyday practice. Therefore, the generation of science based guidelines for proper cleaning practices of AMCs is one of the important tasks for COST AMiCI group as well as the whole scientific audience.

A balanced risk-benefit analysis of AMCs applications should be assessed to guide SbD development addressing (i) mechanisms of action of (nano)-coatings during life-cycle; (ii) quality, efficacy and resistance issues; (iii) safety requirements during use in different applications, procedures and product. There is urgent need for creation of a set of guidelines for all relevant stakeholders (e.g., standards, regulations, recommendations).

Current challenges of SbD approach in the development of innovative AMCs include: (i) establishment of a representative field or field simulation test to describe the actual performance of coatings; (ii) standardized method and/or approaches for a risk-benefit assessment of different AMC in different healthcare environments; (iii) identification of the release of (active) ingredients from the coatings into the environment; (iv) prediction of resistance or cross-resistance invoked by AMCs.

For successful SbD design of novel AMCs, there is an urgent need for predictive models to assess the development of AMR, appropriate (end use dependent) test methods that are standardized and validated methods for QES assessment of AMCs together with appropriate certified reference materials, clear recommendations from regulatory authorities.

Business and industry have a central role in furnishing new affordable materials onto the market with the involvement of all methodologies and professional people shown above, but the final step to be sure that really effective and safe AMCs are adopted is of course the task of the hospitals and other end-users of these AMCs as well as regulatory organizations. Involvement of medical doctors and microbiologist is important especially at the pilot-scale level for the discussion about the effectiveness of AMCs and possible adverse-effects in the clinical environment.

Although there have been studies examining AMCs in healthcare settings, most of them are of insufficient quality and no conclusive findings on beneficial effects on use of AMCs on decrease of HCAI can be retrieved. Dancer [106] clearly suggested that more work is required on the AMCs as futuristic antiseptic surfaces.

To weigh risks versus benefits for NMs over the nanoproducts life cycle is especially difficult task for small and medium sized enterprises (SMEs) that lack the knowledge and resources to conduct this assessment properly. These challenges have been addressed by introducing LICARA nanoSCAN—a modular web based tool for assessing benefits and risks associated with new or existing nanoproducts [107]. This tool is comparing both the benefits and risks over the nanoproducts life cycle. Importantly, the comparison is made with a reference product with a similar functionality. The risks are assessed for public, workers and consumers whereas the benefits with the use of the product are assessed for economy, environment and society. The LICARA nanoSCAN was tested by SMEs and one of the case studies was an antibacterial nanosilver coating for door handles in hospitals. The in-depth assessment concluded that ’*The socio-economic benefits of infection prevention are very high compared to the risks of the NM, although LICARA nanoSCAN somewhat underestimated the respective socio-economic benefits*’ [107]. By applying this tool, the risks can be assessed for public, workers and consumers whereas the benefits with the use of the product are assessed for economy, society and the environment [107]. Thus, the risks-benefit analysis should be conducted pro-actively.

## 5. Conclusions

This paper intended to provide a comprehensive view on different aspects of (eco)toxicological risks and potency to induce AMR resulting from the use of AMCs in healthcare sector. These issues are discussed in light of recently published scientific literature and expert views of the members of COST Action AMiCI.

Weighing the beneficial and adverse effects of AMC in healthcare settings requires the thorough assessment on the following topics using multidisciplinary and multilevel approaches:

- Ecotoxicological hazard needs to be evaluated proactively, before the use of AMCs in healthcare settings’ surfaces in the environment of patients
- The lessons learnt in AMR should be taken on board when assessing the risks of AMCs
- The quality, efficacy and safety evaluation of antimicrobial materials in healthcare settings should be addressed at the level of safe-by-design approach
- Involvement of concerned stakeholders in the risk-benefit analysis is important for the responsible development of AMCs.

## Figures and Tables

**Table 1:** Median LC50 or EC50 for selected aquatic organisms and median MIC for bacteria for Ag, CuO and ZnO NPs summarised from the scientific literature. In the brackets next to the median value the number of data used to derive the median value is presented. Table is modified from [35] with permission of authors.

Group of Organisms/Toxicity Endpoint	Median L(E)C50 ** or MIC *, on Compound Basis, mg/L		
	Ag NPs	CuO NPs	ZnO NPs
Crustaceans (LC50) **	0.01 (17)	2.1 (8)	2.3 (10)
Algae (EC50) **	0.36 (17)	2.8 (5)	0.08 (5)
Fish (LC50) **	1.36 (17)	100 (1)	3.0 (4)
Bacteria (MIC) *	7.10 (46)	250 (13)	622 (15)
Lowest L(E)C50, MIC	0.01	2.1	0.08
Most sensitive organisms	crustaceans	crustaceans	algae

* MIC—minimal inhibitory concentration; ** L(E)C50—half-lethal or half-effective concentration.

**Table 2:** A set of key issues relevant for SbD approach.

Issue	Description	Need
Identification/characterisation of NM-based biocidal agent	Knowledge on the key characteristics that influence the release, exposure, behaviour, effects and subsequent environmental and human risks of NMs.	Reasonably priced, accessible, standardized and validated methods and procedures to characterize NM in different media according to the EC definition.
Transformation of NM-based biocidal agent	Knowledge on the circumstances, extent and rate of dissolution; change of the structure of NM throughout the different stages of their life cycle.	Life Cycle Assessment in different biological and environmental matrices; standardized and validated methods to test or predict the extent and rates of the transformation of NMs.
Dose metrics	Dose that determines a particular response in a test system; production volume of the substance; dose levels at which toxicity effects are observed in experimental tests and which can be compared to the estimated exposure levels to estimate the risk.	Development and use of standardized protocols for sample preparation and characterization of NM within exposure and toxicity studies; identification of the most appropriate metrics for each type of NM within each specific route of exposure and toxicological endpoint.
Extrapolation	Information (on physico-chemical characteristics, exposure and/or hazard) of different forms, types and sizes of NMs (or the bulk material) for extrapolation, read across or grouping within the risk assessment of NMs.	Development of nano-specific approaches for extrapolation, interpolation, read across and grouping based on the key characteristics/properties that influence the release, exposure, behaviour (fate and kinetics), effects (hazards) and subsequent risks of NMs.
Fate and kinetics	Interaction of NMs with their environment that change their physico-chemical characteristics, including their surface composition, ability to aggregate, agglomerate and/or dissolve.	Knowledge on the key characteristics that influence the fate, behaviour and kinetics of NM with respect to the life cycle assessment of nanoproducts (including the release of NMs from products).

## Abbreviations

AMC	antimicrobial coating
AMR	antimicrobial resistance
HCAI	HealthCare Associated Infection
NM	nanomaterials
NP	nanoparticle
SbD	Safe-by-Design
